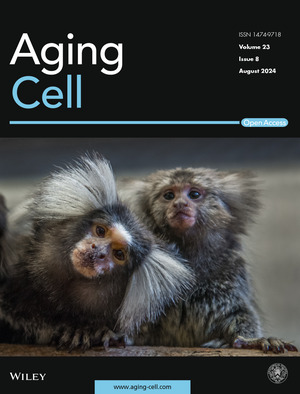# Featured Cover

**DOI:** 10.1111/acel.14331

**Published:** 2024-08-31

**Authors:** Marcus Dittrich, Laura Bernhardt, Christopher A. Penfold, Thorsten E. Boroviak, Charis Drummer, Rüdiger Behr, Tobias Müller, Thomas Haaf

## Abstract

Cover legend: The cover image is based on the Article *Age‐related and species‐specific methylation changes in the protein‐coding marmoset sperm epigenome* by Marcus Dittrich et al., https://doi.org/10.1111/acel.14200.